# Preexisting Immunity to Pandemic (H1N1) 2009

**DOI:** 10.3201/eid1511.090685

**Published:** 2009-11

**Authors:** Zheng Xing, Carol J. Cardona

**Affiliations:** University of California, Davis, CA, USA (Z. Xing, C.J. Cardona); Medical School, Nanjing University, Nanjing, People’s Republic of China (Z. Xing)

**Keywords:** Influenza, immunity, influenza A pandemic (H1N1) 2009 virus, epitopes, viruses, expedited, letter

**To the Editor:** The influenza A pandemic (H1N1) 2009 virus contains a combination of 8 gene segments (*1*–*3*) antigenically similar to North American influenza A virus (H1N1) but different from seasonal human influenza A viruses (H1N1) (*3*). Despite the initial high number of deaths among patients in Mexico and among patients with specific preexisting conditions, pandemic (H1N1) 2009 virus in general has caused mild symptoms, and the overall death rate remains around 0.45% (www.who.int/csr/don/2009_07_06/en). Low virulence of the virus and preexisting immune status are among the main factors that account for lower death rates in influenza outbreaks. The Centers for Disease Control and Prevention (Atlanta, GA, USA) reported that among persons >60 years old, 33% have preexisting, cross-reactive neutralizing antibodies against the new virus, but seasonal influenza vaccines do not elicit cross-reactive neutralizing antibodies against pandemic (H1N1) 2009 virus in either younger or older populations (*1*). However, current data cannot be used to evaluate the full immune capacities of human populations because cell-mediated immunity (CMI) has not been characterized in humans infected with pandemic (H1N1) 2009 virus.

We performed a survey (*4*) for known human immune epitopes present in the various proteins of seasonal influenza A virus strains and known to be efficient in stimulating lymphocytes. We found that multiple major histocompatibility complex (MHC)–restricted epitopes are conserved in nucleoprotein (NP) and matrix protein (MP), and even a few in the more variable hemagglutinin (HA) protein, in A/California/04/2009, A/Texas/04/2009, and A/New York/18/2009.

For MHC class II antigen-restricted epitopes essential for antibody and Th1 responses, HA of pandemic (H1N1) 2009 virus contains HLA-DRA*0101/DRB1 *0101-restricted SVIEKMNTQFTAV (*5*), as well as HLA-DRA*0101/DRB1*0401-restricted EKMNTQFTAVGKE, TGLRNIPSIQSRG, and ELLVLLENERTLDY (*5*), and HLA-DRB5*0101-restricted DYEELREQLSSVSSFERFE (*5*) epitopes. These antigen-restricted epitopes were present in globally-distributed seasonal H1N1 viruses, including classical A/New Caledonia/20/1999 (H1N1) and A/Solomon Islands/3/2006 (H1N1). Overall, high levels of cross-reactive microneutralization (MN) or hemagglutination inhibition (HI) antibodies may not be detected against pandemic (H1N1) 2009 virus. This lack of detection of MN or HI antibodies is probably because most of these epitopes may not elicit MN/HI detectable antibodies, or these epitopes may be present in earlier seasonal influenza strains but not present in the current trivalent, inactivated influenza vaccine. Even though many do not contribute to neutralizing antibodies, these MHC class II antigen-restricted epitopes may initiate the Th1 response, including activation of infected macrophages and antiviral cytokine production, and help host defenses as well.

For MHC class I antigen-restricted epitopes essential for CD8+ T cell activation and CMI, HA of pandemic (H1N1) 2009 virus contains HLA-A*0201-restricted GLFGAIAGFI (*6*), which is present also in the HA of A/New Caledonia/20/1999 (H1N1) and A/Solomon Islands/3/2006 (H1N1) viruses. More MHC class I antigen-restricted epitopes in NP and MP of seasonal epidemic influenza viruses (H1N1) and (H3N2) are conserved in pandemic (H1N1) virus. These seasonal influenza viruses were isolated in North America, Europe, Africa, and Asia–Pacific regions. Conserved epitopes in the HA and NP of pandemic (H1N1) 2009 virus are listed in the Table. In addition, ≈15 completely conserved epitopes are in the M protein of pandemic (H1N1) 2009 virus (data not shown).

Studies have demonstrated that both humoral and cell-mediated immune responses may contribute to protection in influenza-vaccinated persons. As for humoral immunity, results have consistently indicated that serum HI or MN antibody titers correlate inversely with morbidity rates after vaccination, which are the most valuable correlates of protection (*7*). Studies supporting the role of CMI in influenza viral clearance and host survival are well-documented in mouse models, but data are limited for humans. However, emerging evidence has demonstrated that either infection or vaccination can induce T cell-mediated immune responses in humans (*8*,*9*). Moreover, higher levels of CD8+ T cells correlate with reduced viral shedding among experimentally infected humans (*10*). Notably, among vaccinated persons >60 years of age, measures of the ex vivo cellular immune response are statistically correlated with protection against influenza illness but serum HI antibody levels are not, suggesting a role for CMI (*9*). Therefore, it is rational to expect that CMI does provide a protective role, and cross-reactive CMI to pandemic (H1N1) 2009 virus through conserved MHC class I-restricted epitopes may exist in persons previously vaccinated for or exposed to seasonal influenza.

We note that ≈80% of MHC class I epitopes in NP of seasonal and flu vaccine viruses (Table) are also completely conserved in the highly pathogenic avian H5N1 virus (A/Hong Kong/156/1997 and A/Hong Kong/97/1998) (www.ncbi.nlm.nih.gov/genomes/FLU). Several points have to be made regarding the relevance of these epitopes to its high associated mortality rate. First, influenza virus (H5N1) is known to be highly virulent, replicating at a much faster pace than other influenza A viruses and spreading in vital organs shortly after infection and before epitope-mediated protective immunity can be launched, which may account for its high fatality rate. Second, the epitopes are MHC class I antigen-restricted, which means that only a fraction of the human population will possess the correct MHC class I molecules capable of presenting a specific epitope and eliciting appropriate and protective CMI responses. This lack of correct MHC class I molecules could explain why patients of varied genetic backgrounds may have different prognoses upon infection with pandemic (H1N1) 2009 virus or even influenza virus (H5N1).

**Table:** Conserved MHC class I antigen-restricted epitopes present in HA and NP proteins of pandemic (H1N1) 2009 virus*

MHC antigen	Epitope position	Sequence	Identified in selected virus isolates
HA			
HLA-A*0201	344–353	GLFGAIAGFI	A/New Caledonia/20/1999 (H1N1) A/Solomon Islands/3/2006 (H1N1) A/Macau/229/2008 (H1N1) A/Managua/254.01/2008(H1N1
NP			
HLA-A1	44–52	CTELKLSDY	A/Hong Kong/HKU4/2004 (H3N2)
HLA-A*0101			A/Canterbury/200/2004 (H3N2)
HLA-A3	265–273	ILRGSVAHK	A/New Caledonia/20/1999 (H1N1)
			A/New York/55/2004 (H3N2)
			A/Managua/254.01/2008(H1N1)
			A/Taiwan/2645/2006 (H1N1)
HLA-B27	380–393	ELRSRYWAIRTRSG	A/New Caledonia/20/1999 (H1N1)
			A/Taiwan/2645/2006 (H1N1)
			A/Managua/254.01/2008(H1N1)
			A/Florida/UR06-0383/2007(H1N1)
HLA-B27	174–184	RRSGAAGAAVK	A/New Caledonia/20/1999 (H1N1)
			A/New York/55/2004 (H3N2)
			A/California/UR07-0067/2008 (H3N2)
			A/Taiwan/2645/2006 (H1N1)
HLA-B*2705	357–370	KLSTRGVQIASNEN	A/New Caledonia/20/1999 (H1N1)
			A/New York/55/2004 (H3N2)
			A/Hong Kong/HKU77/2005 (H3N2)
			A/Managua/3153.01/2008 (H1N1)
HLA-B*2705	383–391	SRYWAIRTR	A/New Caledonia/20/1999
			A/Taiwan/2645/2006 (H1N1)
			A/Managua/4537.03/2008 (H1N1)
			A/Florida/UR07-0026/2008 (H1N1)
HLA-B*2702	381–388	LRSRYWAI	A/New Caledonia/20/1999
			A/Florida/UR06-0383/2007(H1N1)
HLA-B*4002	251–259	AEIEDLIFL	A/Canterbury/200/2004 (H3N2)
			A/Hong Kong/HKU71/2005 (H3N2)
HLA-B8	225–233	ILKGKFQTA	A/New Caledonia/20/1999
			A/California/UR07-0067/2008 (H3N2)
			A/Taiwan/2645/2006 (H1N1)
HLA-B8	380–388	ELRSRYWAI	A/New Caledonia/20/1999 (H1N1)
HLA-B*0801			A/Taiwan/2645/2006 (H1N1)
			A/Managua/107.01/2008 (H1N1)
			A/Florida/UR07-0026/2008 (H1N1)

*HA, hemagglutinin; NP, nucleoprotein; MHC, major histocompatibility complex; NA, neuraminidase. Epitope binding to and/or activation of specific lymphocytes prepared from human peripheral blood mononuclear cells (PBMC): MHC-tetramer staining; T-cell receptor binding; ELISPOT and intracellular cytokine staining (interferon γ), and/or ^51^Chromine release and killing have been demonstrated in published studies. Data on epitope characterization were collected from the Immune Epitope Database (IEDB; www.immuneepitope.org) (*4*).

In fact, although there are no experiments establishing a solid link, cross-reactive immunity from seasonal influenz or vaccination may result in partial protection of patients infected with influenza virus (H5N1). As reported by WHO for influenza virus (H5N1)–infected patients, the incidence of reported infections was lower for those >40 years of age (22/202, 10.9%) than for those <39 years of age (180/202, 89.1%), and the fatality rate was 32% (7/22) for those >40 years of age and 59% (106/180) for those <40 years of age from 2003 to 2006 (www.who.int/wer/wer8126.pdf). Therefore, repeated exposure to seasonal influenza viruses or vaccination may have resulted in partial cell-mediated or humoral immunity to influenza virus (H5N1). The same type of immunity may have happened in persons exposed to pandemic (H1N1) 2009 virus as well.
